# Serum 25-hydroxyvitamin D and metabolic syndrome: a large cross-section study with dose response analysis in a health screening population

**DOI:** 10.3389/fnut.2026.1809892

**Published:** 2026-04-30

**Authors:** Jia He, Ying-Yun Peng, Xiao-Kang Zhou, Yi-Na Shao, Hua-Fang Du, Hai-Zhen Wang, Tai-Wei Ye, Ying-Gang Chen

**Affiliations:** 1 Department of Health Management Center, Affiliated Jinhua Hospital, Zhejiang University School of Medicine, Jinhua, Zhejiang, China; 2 Department of Colorectal and Anal Surgery, Affiliated Jinhua Hospital, Zhejiang University School of Medicine, Jinhua, Zhejiang, China

**Keywords:** dose–response relationship, health screening population, metabolic syndrome (MetS), restricted cubic spline analysis, serum 25(OH)D

## Abstract

**Background:**

The association between serum 25-hydroxyvitamin D [25(OH)D] and metabolic syndrome (MetS) remains controversial, particularly in large-scale health-screening populations where early metabolic abnormalities can be detected. This study aimed to investigate the independent and potential non-linear association between serum 25(OH)D levels and MetS in adults undergoing routine health examinations.

**Methods:**

This cross-sectional study included 29,214 adults who underwent routine health examinations at a tertiary hospital between January 2024 and November 2025. MetS was defined according to the 2023 Chinese expert consensus criteria. Multivariable logistic regression models with sequential adjustment were used to estimate odds ratios (OR) and 95% confidence intervals (CI) across quartiles of serum 25(OH)D. Restricted cubic spline (RCS) analysis was performed to explore potential non-linear dose–response relationships. Sensitivity analyses included exclusion of outliers, additional adjustment for hepatic and renal function indicators, and *E*-value estimation to assess robustness to unmeasured confounding.

**Results:**

Among 29,214 participants, 6,002 (20.5%) were diagnosed with MetS. After multivariable adjustment, higher serum 25(OH)D levels were independently associated with lower odds of MetS. Compared with the lowest quartile (Q1, <14.9 ng/mL), participants in the highest quartile (Q4, >25.2 ng/mL) had a 36% lower likelihood of MetS (OR = 0.64, 95% CI: 0.57–0.71). RCS analysis demonstrated an inverse dose–response relationship, with the strongest risk reduction observed above approximately 20 ng/mL.

**Conclusion:**

In this large health screening–based population of 29,214 adults, higher serum 25(OH)D concentrations were independently associated with lower odds of metabolic syndrome after comprehensive adjustment for potential confounders. Although the findings support a robust inverse association, causal inference cannot be established, and residual confounding may remain. Prospective cohort studies and well-designed randomized trials are warranted to clarify whether vitamin D plays a causal role in the development of metabolic syndrome or primarily reflects underlying metabolic health status.

## Introduction

With the evolution of social lifestyles, global public health priorities have shifted from isolated disease entities toward complex illnesses and systemic syndromes. Taking obesity—particularly central obesity—as an example, early manifestations may include dysregulated glucose metabolism, elevated blood lipids, or impaired blood pressure control, which can progressively develop into broader metabolic disturbances. In essence, metabolic syndrome (MetS) is a cluster of metabolic disorders characterized by interrelated and interacting comorbidities. Within this syndrome, multiple dysregulations interact, leading to more numerous and severe complications. Because of geographic heterogeneity and variability in diagnostic definitions, the precise worldwide prevalence of MetS is difficult to determine, although current estimates suggest that roughly one in four adults globally are affected (1). Prevalence varies substantially depending on diagnostic criteria and region (2, 3). In China, the prevalence appears to be relatively higher, approaching 30% (4).

Numerous previous studies have demonstrated that individuals with MetS exhibit a significantly higher all-cause mortality rate. This is closely linked to increased cardiovascular risks resulting from dysregulated blood pressure and complications arising from impaired glucose metabolism (1, 5, 6). Therefore, implementing effective health management and early identification of modifiable risk factors in this population represents a critical step in addressing this challenge. Routine health examinations provide a window of opportunity for the early identification and intervention of MetS. Individuals undergoing regular health check-ups are often asymptomatic, yet may already exhibit early metabolic disturbances (7). Identifying modifiable biomarkers associated with MetS in this population is therefore essential for effective risk stratification, early intervention, and the optimization of preventive and nursing management strategies.

Vitamin D deficiency exhibits variations across time, geography, and populations, yet its global average prevalence remains approximately 35.4%. The prevalence increases with higher latitude and is disproportionately higher among women, individuals with obesity, and older adults. According to global epidemiological statistics, nearly one-eighth of the global population has insufficient serum 25(OH)D levels (8, 9). Serum 25(OH)D is the primary biomarker used to assess vitamin D status *in vivo* and has traditionally been recognized for its essential role in bone and mineral metabolism. Beyond skeletal health, accumulating evidence suggests that vitamin D is closely involved in multiple metabolic pathways, including glucose metabolism, lipid regulation, blood pressure control, inflammatory responses, and endothelial dysfunction. Consequently, its potential role in metabolic health has received increasing attention in recent years (10–12).

It has been shown by multiple epidemiological studies that enhanced serum 25(OH)D levels correspond to a lower risk of MetS, and this inverse association is also present in individuals with central obesity and dyslipidemia (13). Furthermore, evidence from dose–response meta-analyses has indicated that serum 25(OH)D concentrations exert an impact on the development of MetS. In particular, individuals with higher concentrations have a significantly reduced risk of developing MetS, and this effect is more pronounced in developed countries, with a risk reduction of up to 43% (14). However, such findings lack validation from prospective epidemiological studies or randomized controlled trials (RCT). Although several previous studies have reported this association, the statistical significance was lost after adjustment for relevant covariates. Differences in study populations, methods of vitamin D categorization, seasonal variation, and liver and renal function may partially account for these discrepancies (15, 16). Therefore, evidence derived from adult health-screening populations that simultaneously account for key confounding factors and rigorously evaluate the robustness of the association remains insufficient. Building upon previous studies, the present study aimed to investigate the relationship between serum 25(OH)D levels and MetS in a health examination cohort from eastern China, with comprehensive adjustment for potential confounders and additional robustness analyses to provide more reliable real-world evidence.

Several biological mechanisms may explain the potential association between vitamin D status and metabolic syndrome. Vitamin D exerts many of its metabolic effects through activation of the Vitamin D Receptor (VDR), which is widely expressed in adipose tissue, pancreatic β-cells, and skeletal muscle and plays an important role in lipid and glucose metabolism (17). Activation of the VDR has been reported to enhance insulin secretion and improve insulin sensitivity while reducing chronic low-grade inflammation. In addition, vitamin D may regulate blood pressure by suppressing the Renin–Angiotensin–Aldosterone System (RAAS) and improving endothelial function (18). Vitamin D may also influence adipokine secretion and inflammatory pathways, which are key processes involved in the development of metabolic syndrome (19).

This cross-sectional study aimed to investigate the independent association between serum 25(OH)D levels and MetS in a health-screening population from eastern China. To achieve this objective, multivariable-adjusted models were applied to control for potential confounding factors. Potential non-linear relationships were further explored using RCS analyses. In addition, several sensitivity analyses were conducted, including exclusion of extreme values, additional adjustment for hepatic and renal function indicators and the timing of blood sampling, as well as E-value estimation to assess the robustness of the findings and the potential influence of unmeasured confounding.

## Methods

### Study design and population

This cross-sectional study was conducted at the Health Management Center of Jinhua Central Hospital and followed the Strengthening the Reporting of Observational Studies in Epidemiology (STROBE) guidelines. Individuals who underwent health examinations at the Health Management Center of Jinhua Central Hospital between January 1, 2024, and November 30, 2025, were consecutively enrolled for personal health monitoring or routine screening. The health examination program included collection of general medical history, measurement of basic anthropometric parameters, and laboratory tests performed by trained medical personnel. A total of 41,777 individuals with recorded serum 25(OH)D measurements were initially assessed for eligibility.

Legally adult individuals were enrolled as the primary study participants. Additionally, all vitamin D–related data were uniformly tested and provided by our center, and individuals included in the study were required to have a complete dataset of indices associated with MetS. Participants were excluded if they met any of the following criteria:

(1) Age<18 years;
(2) Missing key variables required for the diagnosis of MetS or lacking valid personal identification information;
(3) Pregnancy or lactation;
(4) A history of severe liver disease, renal disease, or malignant tumors;
(5) Unreliable laboratory measurements due to compromised blood sample quality;
(6) Participants with multiple health examinations during the study period, for whom only the first examination was retained to avoid repeated measurements and potential clustering effects.

**Figure 1:**
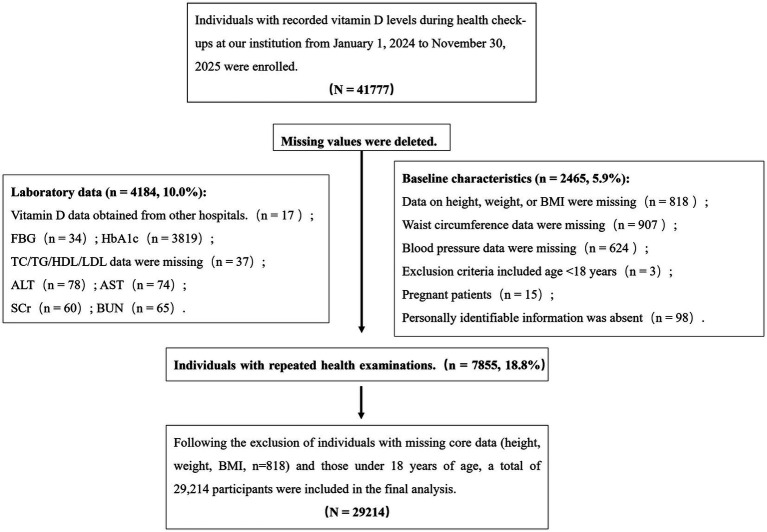
The selection process of the study participants is detailed in the flowchart.

After applying the exclusion criteria, 29,214 participants were included in the final analysis (Figure 1).

The entire study process, from the research protocol to the study outcomes, was conducted in strict accordance with the Declaration of Helsinki and was reviewed and approved by the Ethics Committee of Jinhua Central Hospital, with informed consent waived for this study (20). Given that this research entailed secondary analysis of an existing health examination database, involved no additional interventions or potential risks to participants, and all data were de-identified before the analytical process, the ethics committee granted a waiver for the requirement of informed consent.

### Data collection and measurements

#### Anthropometric measurements

All anthropometric assessments were conducted by trained nurses at the health examination center following standardized operating procedures. Height and body weight were measured using an automatic measuring device (Omron, Japan), and the body mass index (BMI). Notably, height was measured as barefoot net height with shoes removed, and BMI was computed in accordance with international standards, defined as body weight (kg)/height (m^2^). Waist circumference was measured by trained nurses from the health examination center using a non-elastic measuring tape, with participants required to be in a fasting state. The measurement was taken at the midpoint between the palpable inferior costal margin and the superior iliac spine, with participants in a standing position and breathing normally. Blood pressure was measured using an Omron automatic electronic sphygmomanometer (Model HBP9030, Japan), after participants had rested quietly for 5–15 min prior to the measurement.

#### Biochemical measurements

For the collection of biochemical data, participants were required to undergo an overnight fast of at least 8 h, and venous blood samples (a minimum of 5 mL) were collected from them between 6:30 and 9:30 the following morning. All blood samples for biochemical testing were uniformly sent to the Department of Laboratory Medicine, and the relevant assays were performed by physicians specifically responsible for biochemical testing of health check-up samples. A fully automated biochemical analyzer (Beckman Coulter, USA; model AU5800) was utilized to determine various biochemical indicators, including fasting blood glucose (FBG, hexokinase assay), triglycerides (TG, enzymatic assay), total cholesterol (TC, enzymatic assay), high-density lipoprotein cholesterol (HDL-C, direct assay), low-density lipoprotein cholesterol (LDL-C, direct assay), serum creatinine (SCr, Jaffe assay), and blood urea nitrogen (BUN, urease ultraviolet rate assay), along with other relevant biochemical parameters.

Serum 25(OH)D concentrations were expressed as nanograms per milliliter (ng/mL), and the assay was performed via the chemiluminescence method using the Maglumi X8 analyzer (Shenzhen, China). All laboratory analyses were conducted in accordance with standardized operating procedures, and internal quality control was routinely performed throughout the study period.

### Definition of metabolic syndrome

As the study population consisted entirely of Chinese participants, the diagnostic criteria for MetS were adopted from the 2023 Chinese Guidelines for the Prevention and Treatment of Metabolic Syndrome in Adults. This Chinese version differs from the 2006 criteria of the International Diabetes Federation in the waist circumference threshold for females (21, 22). Central obesity, defined as a waist circumference ≥90 cm in men and ≥85 cm in women, was regarded as an essential component for the diagnosis of MetS. In addition, the diagnosis could be made if any two of the following criteria were met:

(1) Abnormal glucose metabolism: fasting blood glucose (FBG) ≥ 6.1 mmol/L, 2-h postprandial blood glucose (2hPG) ≥ 7.8 mmol/L, a previous diagnosis of diabetes mellitus, or receiving hypoglycemic drug therapy;
(2) Elevated blood pressure: systolic blood pressure (SBP) ≥ 130 mmHg, diastolic blood pressure (DBP) ≥ 85 mmHg, a previous diagnosis of hypertension, or receiving antihypertensive drug therapy;
(3) Abnormal lipid metabolism: triglyceride (TG) ≥ 1.7 mmol/L, and decreased high-density lipoprotein cholesterol (HDL-C) (<1.4 mmol/L in men and <1.3 mmol/L in women).

### Assessment of serum 25(OH)D

Serum 25(OH)D is the predominant circulating form of vitamin D in the human body; therefore, it was set as the primary exposure variable in this study. Serum 25(OH)D was measured by chemiluminescence assay using the Maglumi X8 system (Shenzhen, China) at our center. As the assay results were reported in ng/mL, all subsequent analyses were conducted using this unit.

### Covariates and potential confounders

Based on the metabolic characteristics of vitamin D and findings from previous studies, we identified potential confounding variables *a priori*. These variables included demographic characteristics, anthropometric measurements, metabolism-related biochemical indicators, and other relevant factors that may influence circulating vitamin D concentrations, such as season. Specifically, age and sex were considered fundamental demographic covariates, while BMI was included as a key anthropometric indicator. Metabolic-associated covariates included blood pressure readings, markers of glucose metabolism, and lipid profiles. Furthermore, considering the significant seasonal variations in both health examinations and vitamin D metabolism, this variable was also incorporated into the study. To avoid overadjustment and evaluate the stability of the observed association, these covariates were sequentially introduced into the multivariate regression model in subsequent analyses.

### Statistical analysis

All statistical computations were carried out using IBM SPSS Statistics (version 25.0; SPSS Inc.) and R software (version 4.5.2). After grouping the cohorts, continuous variables were presented as mean ± standard deviation (SD) for normally distributed data and as interquartile range (IQR) for skewed data. Categorical variables were expressed as case number (percentage), i.e., (*n*, %). Statistical tests were selected based on the data characteristics, with the Mann–Whitney *U* test or Chi-square test applied for analysis as appropriate.

Potential outliers in continuous variables were identified using boxplot inspection and the interquartile range (IQR) method. These observations were documented separately but retained in the primary analyses to reflect the real-world distribution of values in the health examination population. Subsequently, to verify the reliability of the study findings, we performed a sensitivity analysis after excluding outliers. The OR and corresponding 95% CI for serum 25(OH)D and related covariates were calculated using logistic regression. Meanwhile, we constructed a series of multivariate models (from Model 1 to Model 4) with sequential adjustment for covariates. To evaluate the influence of covariates on the results, the study examined the association by sequentially adjusting for relevant covariates. Finally, to investigate the nonlinear dose–response relationship between the two, we constructed a RCS model. In addition, we calculated the OR and corresponding E-values to assess the impact of other potential unmeasured confounding factors.

## Results

### Study population and baseline characteristics

A total of 29,214 individuals who underwent health examinations at the Health Management Center of Jinhua Central Hospital between January 1, 2024, and November 30, 2025, were included after the screening process (Figure 1). The baseline characteristics of the study population are summarized in Table 1. Among the included participants, 6,002 individuals (20.5%) met the diagnostic criteria for MetS.

**Table 1:** Baseline characteristics by metabolic syndrome status.

Variables	No (*n* = 23,212)^1^	Yes (*n* = 6,002)^1^	*P*-value^2^	SMD^3^
Age (years)	47 ± 15	54 ± 15	<0.001	0.52
Sex			<0.001	0.36
Male	11,591 (50%)	4,038 (67%)		
Female	11,621 (50%)	1,964 (33%)		
BMI (kg/m^2^)	23.2 ± 3.0	27.5 ± 3.0	<0.001	1.45
Waist circumference (cm)	79 ± 10	94 ± 8	<0.001	1.71
Systolic BP (mmHg)	123 ± 17	139 ± 16	<0.001	0.97
Diastolic BP (mmHg)	75 ± 10	83 ± 11	<0.001	0.81
Triglycerides (mmol/L)	1.29 (0.94, 1.76)	2.23 (1.74, 3.02)	<0.001	0.68
HDL cholesterol (mmol/L)	1.38 (1.20, 1.60)	1.17 (1.02, 1.35)	<0.001	0.74
Fasting blood glucose (mmol/L)	4.93 (4.63, 5.30)	5.60 (5.01, 6.36)	<0.001	0.67
eGFR (mL/min/1.73 m^2^)	101 ± 19	91 ± 17	<0.001	0.55
Blood sampling season			<0.001	0.13
Spring	3,493 (15%)	1,135 (19%)		
Summer	7,221 (31%)	1,606 (27%)		
Autumn	11,421 (49%)	2,931 (49%)		
Winter	1,077 (4.6%)	330 (5.5%)		
Serum 25(OH)D (ng/mL)	20 (15, 25)	20 (15, 25)	0.12	<0.01

^1^Mean ± SD; *n*(%); Median (Q1, Q3).

^2^Welch Two Sample *t*-test; Pearson’s Chi-squared test; Wilcoxon rank sum test.

^3^SMD, standardized mean difference.

Values are presented as mean ± standard deviation, median (interquartile range), or number (percentage), as appropriate.

*P*-values were calculated using Student’s *t*-test, Wilcoxon rank-sum test, or chi-square test, as appropriate.

In comparison to participants without MetS, those diagnosed with MetS were older and had a higher male proportion. They also exhibited higher levels of BMI, waist circumference, systolic and diastolic blood pressure, fasting blood glucose, and triglycerides, but lower HDL-C and eGFR levels. Standardized mean differences (SMD) were calculated to evaluate the magnitude of baseline differences independent of sample size. Anthropometric and metabolic variables, particularly BMI and waist circumference, showed moderate to large standardized differences, whereas seasonal distribution and serum 25(OH)D levels showed minimal imbalance between groups (Table 1).

### Grouping and distribution characteristics

**Figure 2:**
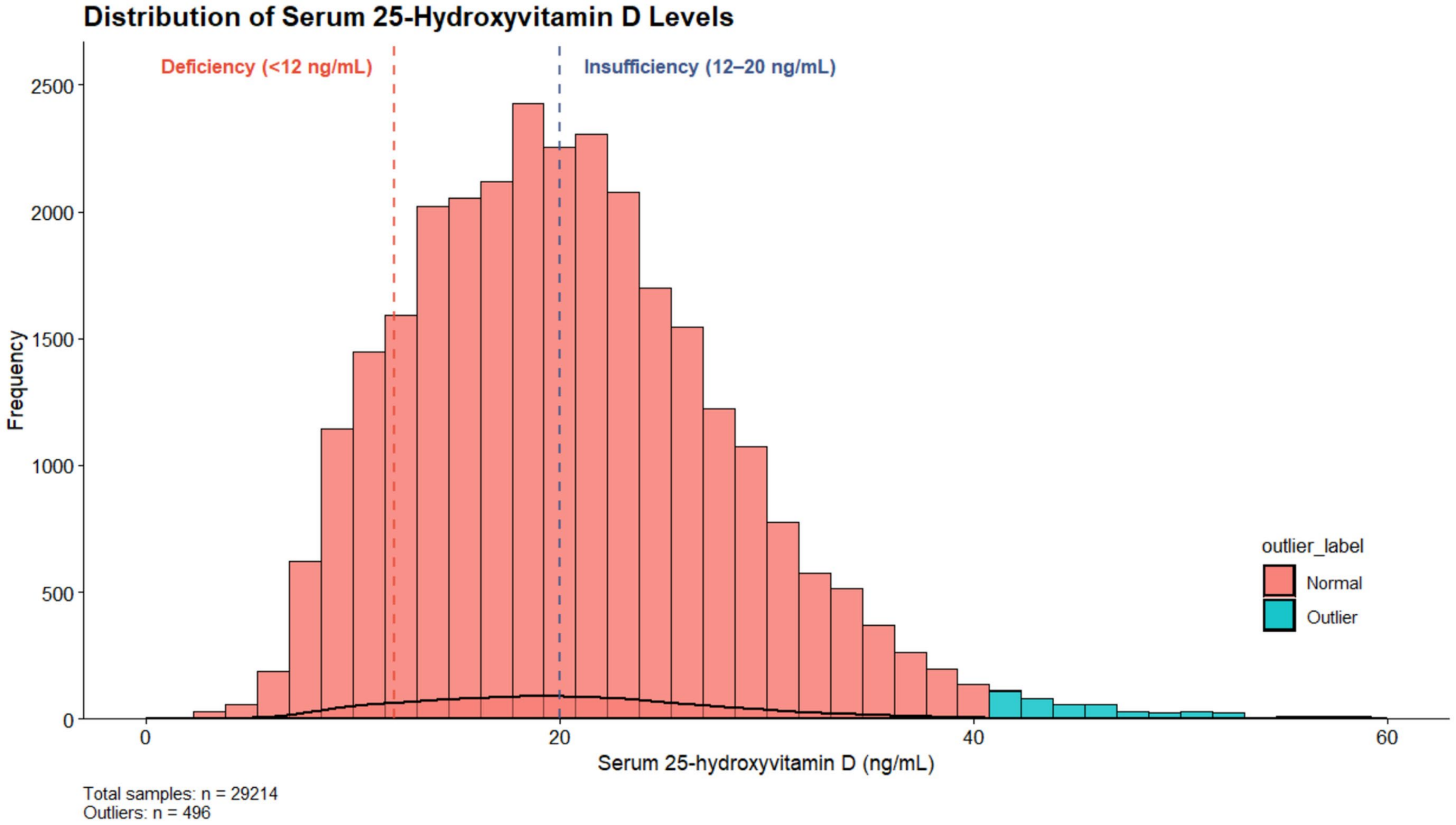
Distribution of serum 25-hydroxyvitamin D levels. Histogram and density plot showing the distribution of serum 25(OH)D levels in the study population. Vertical dashed lines indicate commonly used clinical cut-off values for vitamin D deficiency and insufficiency.

The distribution of serum 25(OH)D levels among participants is presented in Figure 2. The distribution exhibited a slight right skew, with outliers primarily observed in the right tail. The dashed lines indicate the predefined cutoff values for vitamin D deficiency and insufficiency.

**Figure 3:**
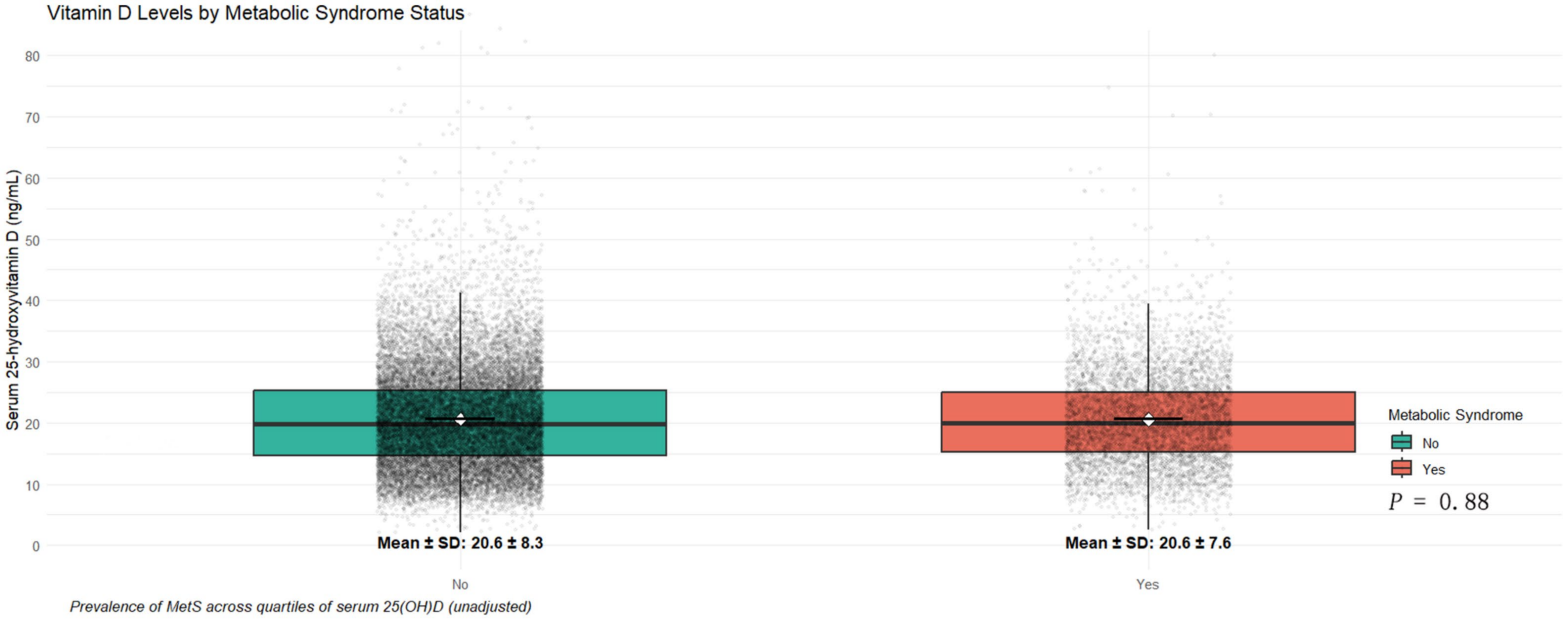
Serum 25(OH)D levels by metabolic syndrome status. Boxplots compare serum 25(OH)D levels between participants with and without metabolic syndrome. Mean ± SD values and unadjusted *p*-values are shown.

The distribution of serum 25(OH)D concentrations in the two groups is presented using a box plot (Figure 3). The mean serum 25(OH)D levels were 20.6 ± 7.6 ng/mL and 20.6 ± 8.3 ng/mL in the two groups, respectively, with no statistically significant difference observed (*p* = 0.88).

The prevalence of MetS across serum 25(OH)D quartiles is summarized in Table 2. The prevalence rates were 18.7% in Q1 (<14.9 ng/mL), 21.8% in Q2 (14.9–19.8 ng/mL), 22.0% in Q3 (19.9–25.2 ng/mL), and 19.7% in Q4 (>25.2 ng/mL). The highest prevalence was observed in Q3, whereas the lowest prevalence occurred in Q1 (unadjusted estimates).

**Table 2:** Prevalence of metabolic syndrome across serum 25(OH)D quartiles.

25(OH)D quartile	Total (n)	MetS cases (n)	Prevalence (%)
Q1	7,350	1,371	18.7
Q2	7,268	1,585	21.8
Q3	7,308	1,610	22.0
Q4	7,288	1,436	19.7
	29,214	6,002	20.5

MetS prevalence is unadjusted.

Serum 25(OH)D quartiles were defined based on the distribution of the study population.

### Multivariable regression analysis

The associations between serum 25(OH)D levels and MetS were evaluated using multivariable logistic regression models (Table 3). In the crude model, an increased odds of MetS was observed in participants in the Q2 and Q3 groups, showing a paradoxical association. This unexpected finding may be explained by confounding, particularly confounding by adiposity. Individuals with higher adiposity are more likely to develop MetS while simultaneously exhibiting lower circulating vitamin D levels due to sequestration of vitamin D in adipose tissue.

**Table 3:** Association between serum 25(OH)D quartiles and metabolic syndrome.

Characteristic	Crude			Model 1			Model 2			Model 3			Model 4 (Fully adjusted)		
	OR	95% CI	*p*-value	OR	95% CI	*p*-value	OR	95% CI	*p*-value	OR	95% CI	*p*-value	OR	95% CI	*p*-value
25(OH)D quartile (Ref: Q1)															
Q1															
Q2	1.22	1.12, 1.32	**<0.001**	0.96	0.88, 1.04	0.3	0.92	0.83, 1.02	0.12	0.92	0.82, 1.02	0.10	0.92	0.83, 1.02	0.13
Q3	1.23	1.14, 1.34	**<0.001**	0.83	0.76, 0.91	**<0.001**	0.77	0.69, 0.86	**<0.001**	0.78	0.70, 0.87	**<0.001**	0.79	0.71, 0.88	**<0.001**
Q4	1.07	0.99, 1.16	0.11	0.57	0.52, 0.63	**<0.001**	0.60	0.54, 0.67	**<0.001**	0.63	0.57, 0.71	**<0.001**	0.64	0.57, 0.71	**<0.001**
Age (per 10 years)				1.04	1.03, 1.04	**<0.001**	1.06	1.05, 1.06	**<0.001**	1.06	1.05, 1.06	**<0.001**	1.06	1.05, 1.06	**<0.001**
Sex (Ref: Male)															
Male															
Female				0.48	0.45, 0.51	**<0.001**	1.24	1.15, 1.35	**<0.001**	1.42	1.31, 1.54	**<0.001**	1.47	1.33, 1.62	**<0.001**
BMI (per 5 kg/m^2^)							1.71	1.69, 1.74	**<0.001**	1.60	1.57, 1.62	**<0.001**	1.60	1.57, 1.62	**<0.001**
Fatty liver															
No															
Yes										3.49	3.18, 3.83	**<0.001**	3.50	3.19, 3.84	**<0.001**
eGFR (per 10 mL/min/1.73 m^2^)													1.00	1.0, 1.00	0.3
Blood sampling season															
Spring															
Summer													0.90	0.80, 1.00	0.057
Autumn													1.01	0.91, 1.12	0.8
Winter													1.03	0.86, 1.24	0.7

CI, confidence interval; OR, odds ratio.

Odds ratios (ORs) and 95% confidence intervals (CIs) were estimated using logistic regression models.

Model 1 was adjusted for age and sex; Model 2 was further adjusted for BMI.

Model 3 was additionally adjusted for fatty liver.

Model 4 was further adjusted for eGFR and season of blood sampling.

Bold values indicate statistical significance (*P* < 0.05).

Accordingly, in Model 1, adjustments were made for demographic variables (sex and age), re-analysis revealed a reversal of the results with a statistically significant negative correlation. Further adjustment for BMI in Model 2 markedly strengthened this inverse association, supporting the hypothesis that adiposity acted as a major confounder in the crude analysis. Additional adjustment for fatty liver status (Model 3) and other covariates (Model 4) produced consistent results, with the highest quartile (Q4) showing a 36% lower risk of MetS compared with Q1 (OR = 0.64, 95% CI: 0.57–0.71, *p* < 0.001). These findings suggest that the apparent paradox observed in the unadjusted model was primarily driven by adiposity-related confounding, and that serum 25(OH)D levels were independently and inversely associated with MetS after appropriate control of confounding factors (Table 3).

### Restricted cubic spline analysis

**Figure 4:**
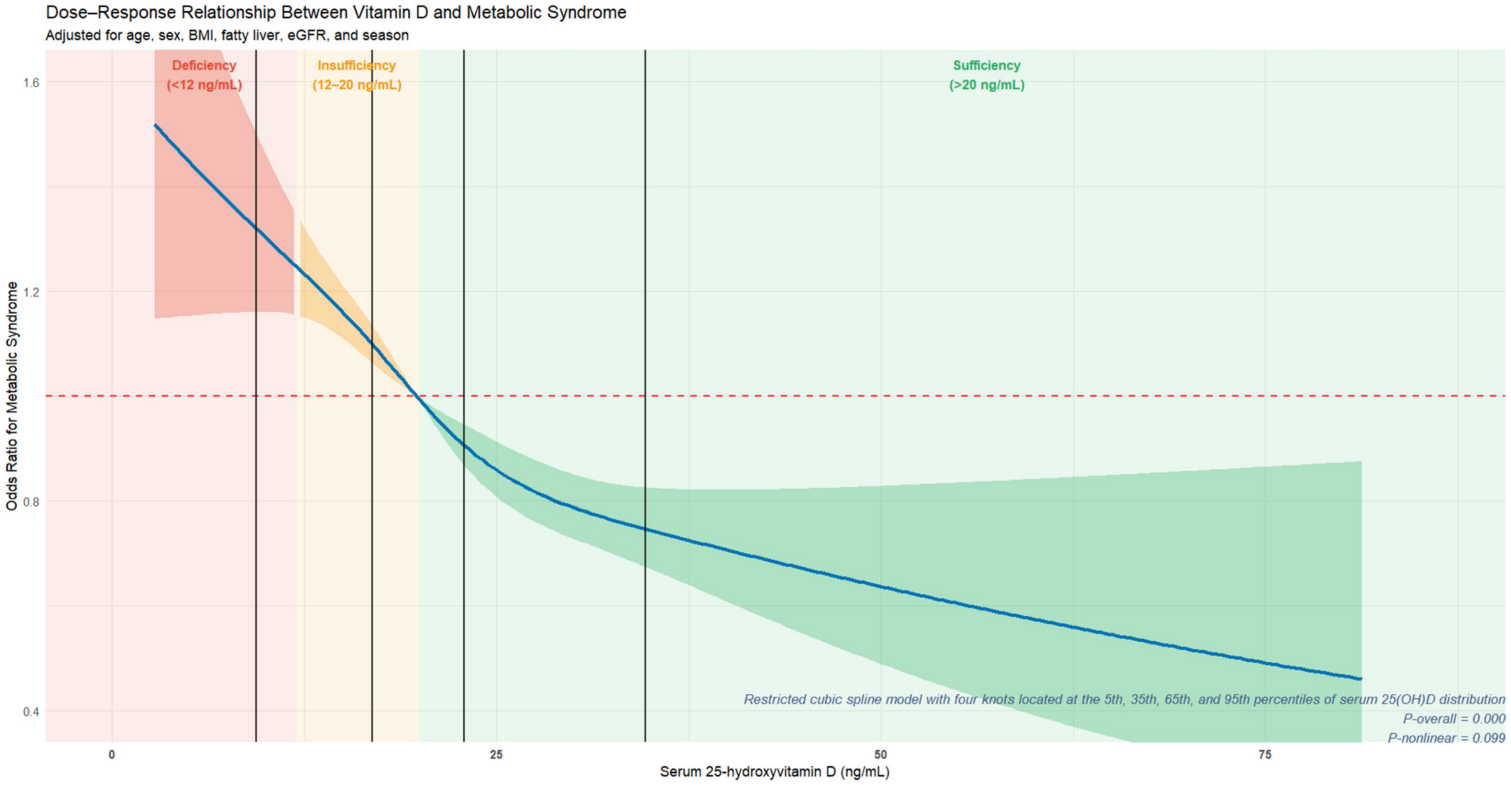
Dose–response relationship between serum 25(OH)D and metabolic syndrome. Restricted cubic spline analysis illustrating the dose–response association between serum 25(OH)D levels and metabolic syndrome. Models were adjusted for age, sex, BMI, fatty liver, eGFR, and season.

The RCS analysis was performed to evaluate the dose–response relationship between serum 25(OH)D concentrations and the risk of MetS, with adjustment for the key covariates described previously. The spline model included four knots placed at the 5th, 35th, 65th, and 95th percentiles of the serum 25(OH)D distribution, and the results are shown in Figure 4. The horizontal red dashed line represents an OR of 1.0, indicating no association between the two variables.

As shown in the RCS curve, the magnitude of reduction in MetS risk was not positively associated with elevated serum 25 (OH) D concentrations. The first half of the curve demonstrated a pronounced reduction in risk, whereas the risk reduction gradually diminished with further increases in concentrations, suggesting a potential nonlinear trend between the two variables. While the curve shape implied a potential non-linear trend, the non-linearity test failed to achieve conventional statistical significance (non-linear *p* = 0.099). Within the range of vitamin D deficiency (<12 ng/mL; red shaded area), the OR for MetS remained consistently above 1.0, indicating that low serum 25(OH)D levels, particularly in individuals with vitamin D insufficiency or deficiency, were associated with a significantly increased risk of MetS. In the insufficiency range (12–20 ng/mL; yellow-shaded area), the OR declined rapidly and crossed the null line (OR = 1.0) at approximately 20 ng/mL. In the sufficiency range (>20 ng/mL; green-shaded area), the OR showed an approximately linear downward trend, reaching around 0.5 at 80 ng/mL, suggesting progressively lower odds of MetS with higher serum 25(OH)D concentrations.

### Outlier and sensitivity analyses

#### Outlier analysis

Outliers identified using the IQR method were assessed across clinical and biochemical variables according to MetS status. Overall, 17.03% of participants exhibited at least one outlier across the assessed variables (Supplementary Table A1). Among all variables, FBG, liver transaminases, including alanine aminotransferase (ALT) and aspartate aminotransferase (AST), showed the highest outlier frequencies, at 7.19, 7.26, and 5.75%, respectively.

The proportions of outliers according to MetS status are presented in Supplementary Figure A1 and Supplementary Table A2. Participants with MetS demonstrated higher proportions of outliers across most variables, particularly for FBG (20.1% vs. 3.8%), ALT (16.8% vs. 4.8%), TG (15.1% vs. 3.2%), and AST (12.8% vs. 3.9%). In contrast, the frequency of serum 25(OH)D outliers was low in both the MetS group (1.3%) and the non-MetS group (1.8%), with no statistically significant difference observed.

#### Sensitivity analysis

First, we compared the OR values for serum 25(OH)D across different quartiles in relation to MetS in both the original dataset (including outliers) and the sensitivity dataset after excluding all outliers (*n* = 24,240). The results are presented in Supplementary Figure A2. A significant negative correlation was observed for serum 25(OH)D in the Q3 and Q4 quartiles, with a high degree of overlap in the OR estimates and their corresponding 95% CI. This finding was consistent with the previous statistical results. Similar patterns were observed after exclusion of extreme values.

Second, we further evaluated the stability of the observed associations by sequentially adjusting for additional potential confounding variables, including eGFR, season of blood sampling, and ALT/AST. The corresponding results are shown in Supplementary Figure A3 and Supplementary Figure A1. The negative dose–response relationship between the two variables remained consistent across all extended multivariate models (Models 3–5). Even after adjustment for all other covariates, the Q4 estimate in Model 5 remained comparable to earlier models.

Third, the odds ratios for MetS across serum 25(OH)D quartiles under progressively adjusted regression models are presented in Supplementary Figure A4. Compared with the reference group Q1, OR estimates for Q3 and Q4 remained below 1.0 across crude and adjusted models, with largely overlapping 95% confidence intervals. The overall distribution pattern of the estimates remained similar after incremental covariate adjustment.

### *E*-value analysis

To further evaluate the potential impact of unmeasured confounding, an *E*-value analysis was performed. For the Q4 versus Q1 comparison, the *E*-value was 1.25 in Supplementary Table A3. This implies that the observed association could only be explained if there existed an unmeasured confounder that was associated with both variables of interest and had an OR greater than 1.25 for the outcome. Although the *E*-value of 1.25 was relatively low, the combined interpretation with the 95% CI (OR = 0.64, 95% CI: 0.57–0.71) indicated that an unmeasured confounder with a substantially stronger effect (OR ≥ 1.82) would be required to explain the observed association. Collectively, these findings demonstrate a moderate level of robustness in the study results to the potential unmeasured confounding factors not included in the analysis (see Supplementary material for detailed sensitivity analyses).

## Discussion

In this large health screening-based cross-sectional study, higher serum 25(OH)D concentrations were independently associated with lower odds of MetS after comprehensive adjustment for potential confounders. However, no causal inferences can be drawn from this finding. Multivariate logistic regression analysis revealed that the negative association persisted after successive adjustment for confounding factors (Table 3). Furthermore, the RCS model was applied to assess the linear relationship between the two variables, and the results indicated a non-linear trend in their association. This indicates that the effect of serum 25(OH)D does not increase uniformly across the entire concentration range, but is more pronounced at lower levels (i.e., <12 ng/mL or 12–20 ng/mL), as shown in Figure 4. To account for potential bias, several sensitivity analyses were conducted. These included exclusion of outliers (Supplementary Figure A2), additional adjustment for liver function, renal function, and blood collection season (Supplementary Table A2), and *E*-value estimation to assess the potential impact of unmeasured confounding (Supplementary Table A3). Across all sensitivity and robustness analyses, the observed association remained stable, supporting the robustness and internal validity of the main findings.

Notably, no statistically significant difference between the two was detected in the unadjusted analysis; however, following multivariable adjustment, serum 25(OH)D levels were significantly inversely correlated with the risk of MetS. This reversal between crude and adjusted models likely reflects substantial confounding by adiposity-related factors. Variables such as BMI and fatty liver status are strongly associated with both serum 25(OH)D concentrations and metabolic risk, potentially inducing a suppression or masking effect in unadjusted analyses. After controlling for these key confounding factors, the independent association between the two became evident (Table 3 and Supplementary Figure A4).

Our results are broadly aligned with the outcomes documented in numerous prior investigations. A recent study conducted in South Korea indicated that each 25 nmol/L increment in serum 25(OH)D was associated with at least a 15% reduction in the risk of MetS (9). In addition, a similar negative correlation has been reported in another observational study, albeit with a slightly smaller reduction in the risk of MetS of approximately 18% (13). Similarly, a population-based study in Rotterdam demonstrated that among elderly individuals with MetS, serum 25(OH)D at high levels correlated with a lower prevalence of MetS, and this finding showed a more significant correlation with individual metabolic components (HDL-C, triglyceride, and blood glucose levels) (23). However, it is noteworthy that not all studies have observed a strict linear association. The non-linear association identified in the current analysis is consistent with the findings reported by Hajhashemy et al. (14). In their study, the greatest reduction in MetS incidence was observed when serum 25(OH)D concentrations were close to the vitamin D insufficiency threshold (approximately 20 ng/mL). Beyond this level, further increases in 25(OH)D were associated with progressively smaller reductions in MetS risk, suggesting a plateau or threshold pattern rather than a strictly linear relationship (13, 14, 24).

Although observational studies have consistently demonstrated an inverse association between the two, these findings remain inconsistent with evidence from randomized controlled trials. A randomized, double-blind RCT reported that 40 participants in the study were randomly assigned to receive a 16-week vitamin D supplementation, with the other group given a placebo. The final results showed a significant reduction in TG levels in the intervention group (*p* < 0.001), while no significant improvements were observed in other study variables. The study concluded that vitamin D supplementation had no beneficial effect on improving major cardiovascular risk factors (25). The discrepancies between the findings of observational studies and randomized controlled trials suggest that serum 25(OH)D is promising as a biomarker or modulator of metabolic risk, rather than a direct etiological factor in the pathogenesis of metabolic syndrome.

Several biological mechanisms may help explain the observed association. Vitamin D, through activation of the VDR, has been shown to upregulate genes involved in cholesterol efflux and fatty acid oxidation, such as *ABCA1* and *CPT-1A*, thereby influencing TC and TG metabolism and promoting HDL-C formation and cholesterol transport. In addition, vitamin D acts on pancreatic β cells and peripheral target tissues to regulate calcium homeostasis, attenuate inflammation and oxidative stress, enhance insulin secretion, and improve insulin resistance (17, 26). Meanwhile, vitamin D exerts multifactorial effects on the maintenance of BP homeostasis. Vitamin D deficiency weakens the inhibitory effect on the RAAS. Meanwhile, it leads to reduced intracellular calcium concentrations and blunted reactivity in vascular smooth muscle, thereby elevating blood pressure. Vitamin D may also contribute to blood pressure regulation through vascular protective effects. It improves endothelial function, inhibits inflammatory mediator production, and alleviates vascular injury, thereby reducing peripheral vascular resistance (18, 19, 27, 28). These anti-inflammatory and vascular effects are likely mediated through interconnected metabolic and endothelial pathways. Overall, vitamin D is unlikely to exert a direct effect on MetS itself but may influence syndrome development indirectly through modulation of its individual metabolic components. Research into the aforementioned mechanisms also supports the existence of a potential negative association between serum 25(OH)D levels and MetS. That said, the inconsistent outcomes from RCT may stem from variations in supplementation dosage, baseline vitamin D status, and heterogeneity across study populations.

Its measurement facilitates the early identification of high-risk individuals who may potentially develop metabolic syndrome, and enables targeted lifestyle modifications and nutritional counseling (29–31). From a clinical perspective, serum vitamin D measurements obtained during routine health examinations may provide useful baseline information for metabolic risk assessment. Based on these measurements, individualized preventive strategies, including dietary modification, guidance on safe sun exposure, and vitamin D supplementation when appropriate, may be implemented. Such strategies may be particularly relevant during periods of reduced outdoor activity or marked seasonal variation in sunlight exposure, which is consistent with our findings after adjustment for seasonal factors (32). Collectively, these findings suggest that serum 25(OH)D may serve as a practical biomarker for early metabolic risk stratification in routine health-screening settings.

A key strength of this research is the systematic evaluation of result robustness. The observed relationships remained consistent across a series of sensitivity analyses. Notably, considering the potential for a large number of outliers in a large sample, we compared the influence on the results before and after outlier exclusion. In fact, the study findings were not confounded by outliers and the expected results were still observed. Furthermore, the association persisted after additional adjustment for liver and renal function as well as season of blood sampling. Lastly, we quantitatively assessed the potential influence of unmeasured confounding. As we noted earlier, although the E-value was relatively small, it still indicated a moderate level of robustness. The application of such quantitative bias analysis remains limited in nutritional epidemiology, yet it can substantially improve the interpretability of observational research (33). Despite the consistency of our findings across multiple sensitivity analyses, the E-value analysis suggests only a moderate degree of robustness to potential unmeasured confounding. Therefore, the influence of residual confounding factors cannot be completely ruled out, and caution should be exercised in the interpretation of the results. Several limitations of the present study can be identified in light of the above. Most importantly, the study design does not allow for establishing causal directionality between the two factors. Second, data on vitamin D supplementation, dietary intake, and sun exposure were lacking; these are all potential unmeasured factors that may have influenced the study outcomes. Finally, the enrolled population consisted predominantly of Chinese participants from a single center, which may limit the generalizability of our findings to other ethnic groups.

In summary, among individuals undergoing routine health examinations, changes in serum 25-hydroxyvitamin D levels are associated with the risk of MetS onset. Future studies should conduct more animal experiments or RCT to validate the plausibility of this relationship, and provide better evidence for decisions regarding vitamin D supplementation in health screening and health management.

## Data Availability

The datasets presented in this article are not readily available due to confidentiality and privacy reasons. Requests to access the datasets should be directed to 411158328@qq.com.
