# Supplementary material for: Serum 25-hydroxyvitamin D and metabolic syndrome: a large cross-section study with dose response analysis in a health screening population
Source: Front Nutr. 2026 Apr 30;13:1809892. doi: 10.3389/fnut.2026.1809892 (PMC13174186; doi:10.3389/fnut.2026.1809892)
Supplement: Supplementary file 1 [file Image_1.PDF]

Figure A1: Heatmap of outlier distribution by metabolic syndrome status.

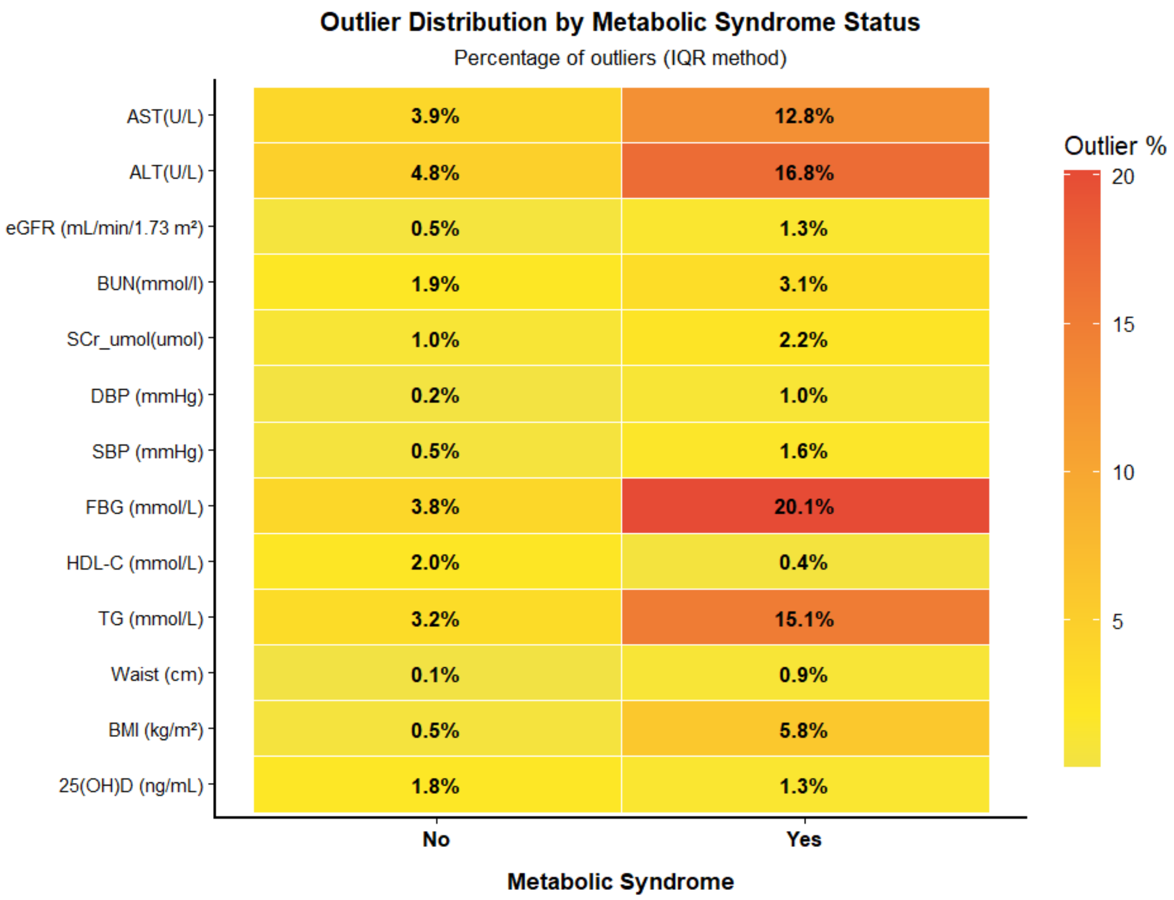

The heatmap illustrates the proportion of outliers for each variable stratified by metabolic syndrome status, highlighting a higher concentration of extreme values among participants with metabolic syndrome.

Figure A2: Sensitivity analysis excluding participants with outliers.

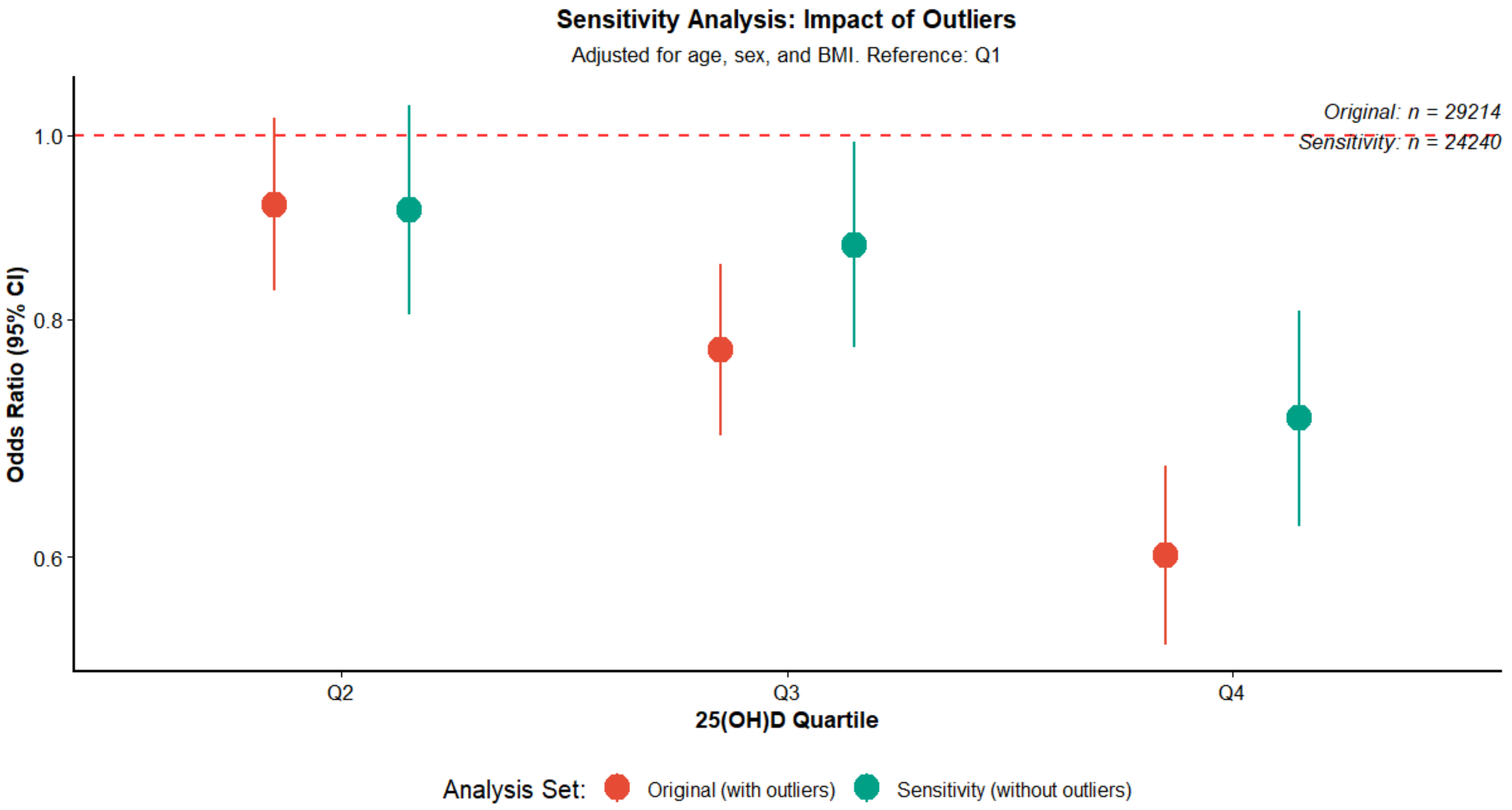

Odds ratios and 95% confidence intervals for metabolic syndrome across serum 25(OH)D quartiles after excluding participants with any identified outliers.

Figure A3: Sensitivity analysis with additional covariate adjustment.

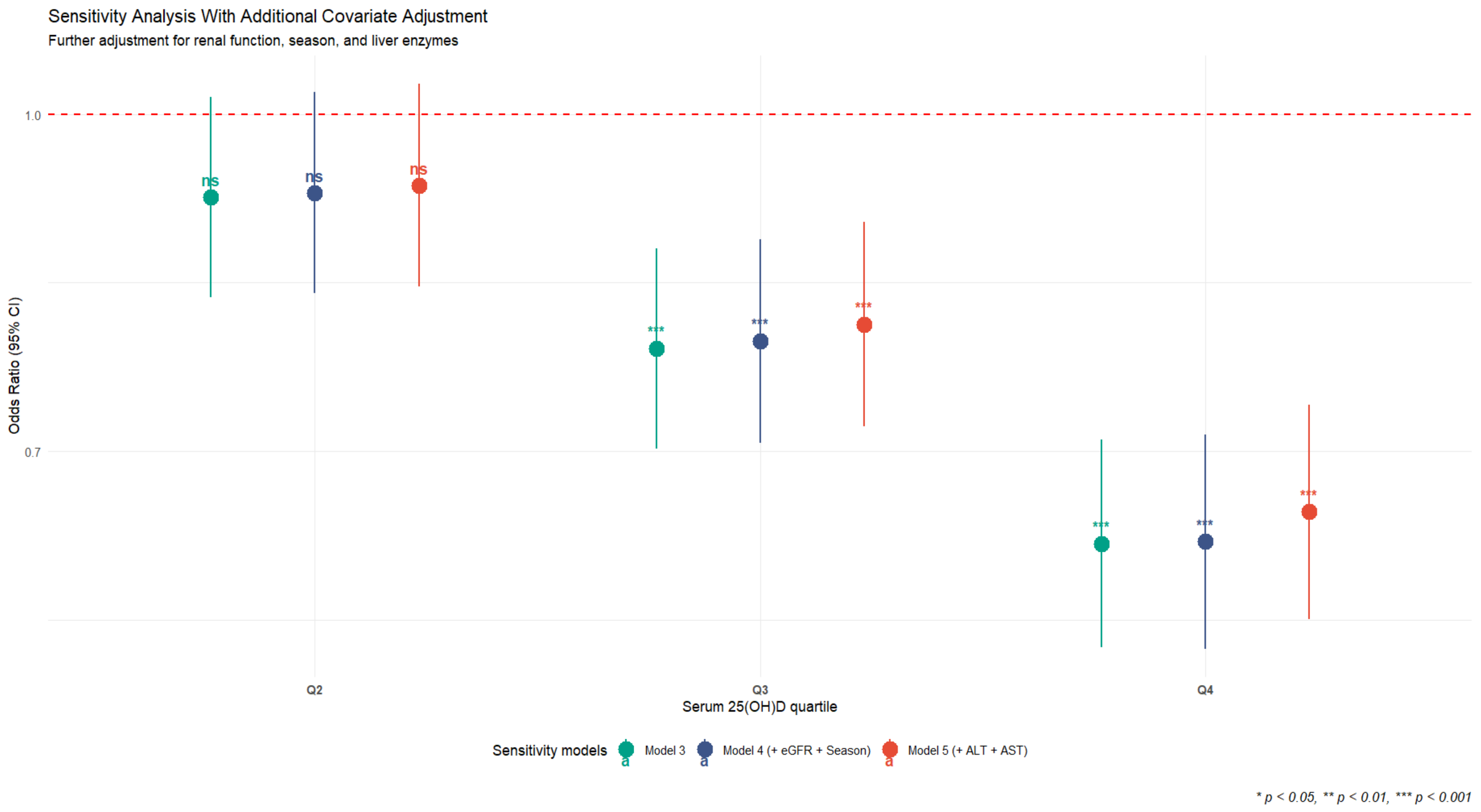

Logistic regression results for metabolic syndrome with further adjustment for additional metabolic and clinical covariates.

Figure A4: Associations between serum 25(OH)D quartiles and the risk of MetS across different adjustment models. (Original Figure 4)

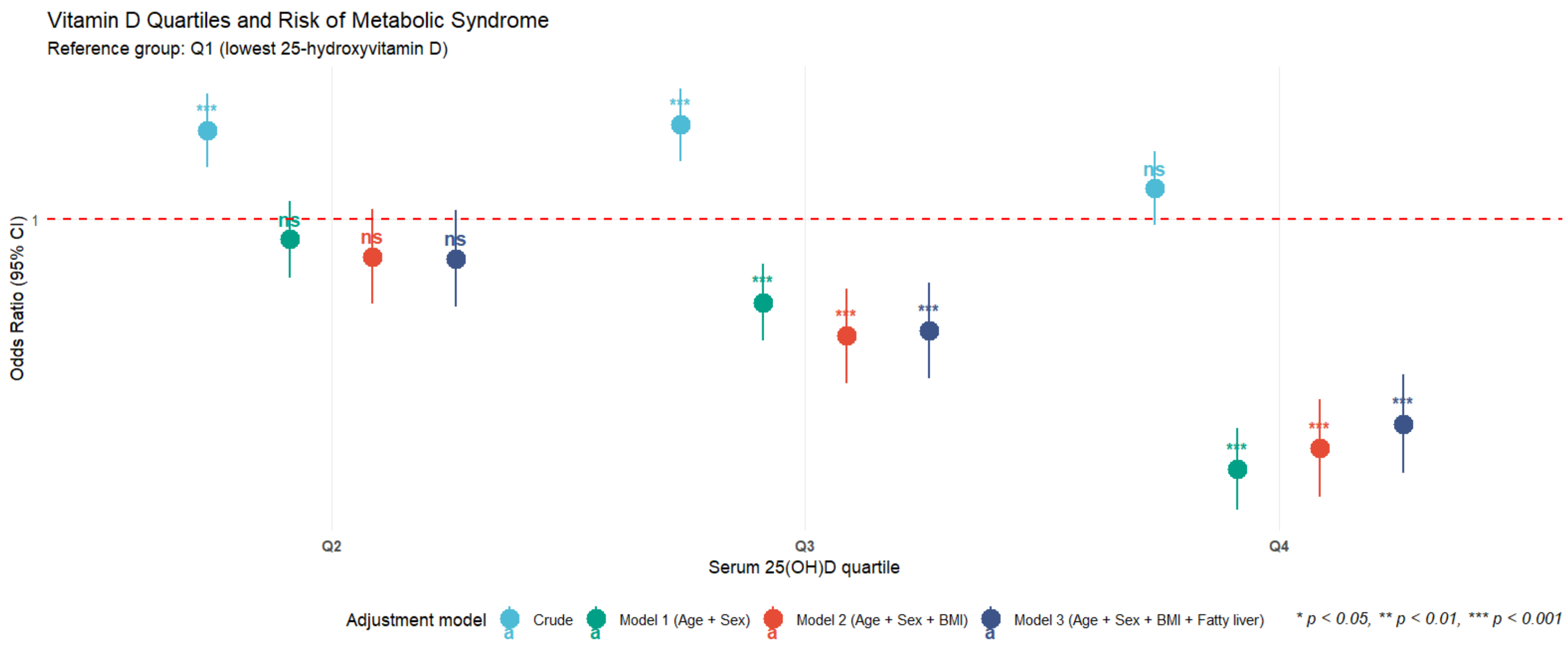

Forest plot displaying odds ratios and 95% confidence intervals for metabolic syndrome across serum 25(OH)D quartiles.(Results are shown for crude and multivariable-adjusted models.)

Figure S1: Sensitivity analysis with additional adjustment for eGFR and season.

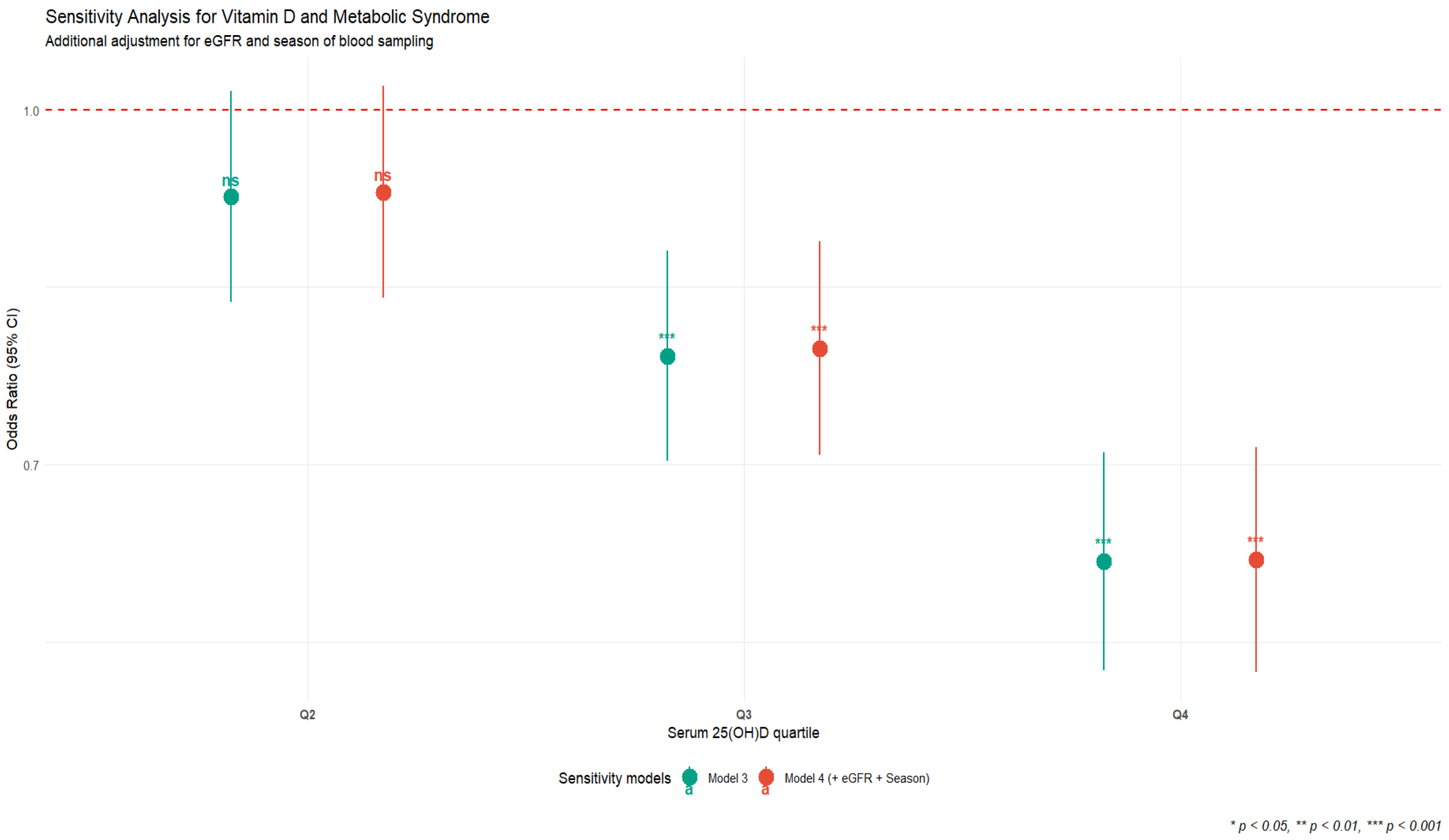

Odds ratios (ORs) and 95% confidence intervals (CIs) for metabolic syndrome across serum 25(OH)D quartiles after additional adjustment for estimated glomerular filtration rate (eGFR) and season of blood sampling.

Results were consistent with the main analysis, supporting the robustness of the association between serum 25(OH)D levels and metabolic syndrome.
