# Supplementary material for: Serum 25-hydroxyvitamin D and metabolic syndrome: a large cross-section study with dose response analysis in a health screening population
Source: Front Nutr. 2026 Apr 30;13:1809892. doi: 10.3389/fnut.2026.1809892 (PMC13174186; doi:10.3389/fnut.2026.1809892)
Supplement: Supplementary file 2 [file Table_1.PDF]

Table A1: Table A1. Summary of outliers identified by the IQR method.

Var Name	Variable	Total (n)	Outliers (n)	Outliers (%)	IQR Lower	IQR Upper	Outlier Min	Outlier Max
VitD_ngml	25(OH)D (ng/mL)	29,214	496	1.70	-0.80	40.80	40.90	119.00
BMI	BMI (kg/m²)	29,214	459	1.57	15.10	32.70	14.40	44.50
Waist	Waist (cm)	29,214	73	0.25	47.50	115.50	115.70	130.00
TG	TG (mmol/L)	29,214	1,658	5.68	-0.60	3.68	3.69	39.18
HDL_C	HDL-C (mmol/L)	29,214	484	1.66	0.53	2.18	0.37	6.47
FBG_mmol	FBG (mmol/L)	29,214	2,100	7.19	3.48	6.68	3.28	23.54
SBP	SBP (mmHg)	29,214	206	0.71	75.88	174.88	175.00	237.00
DBP	DBP (mmHg)	29,214	100	0.34	44.00	108.00	41.00	153.00
SCr_umol	SCr_umol(umol)	29,214	355	1.22	35.50	119.50	120.00	968.00
BUN	BUN(mmol/l)	29,214	628	2.15	1.66	8.46	1.61	46.55
eGFR	eGFR (mL/min/1.73 m²)	29,214	196	0.67	47.83	151.29	4.48	154.73
ALT	ALT(U/L)	29,214	2,122	7.26	-9.75	53.05	53.10	1,865.20
AST	AST(U/L)	29,214	1,681	5.75	5.95	39.95	3.00	2,438.60

Var Name	Variable	Total (n)	Outliers (n)	Outliers (%)	IQR Lower	IQR Upper	Outlier Min	Outlier Max
**out_any**	**Any Variable**	**29,214**	**4974**	**17.03**				

Note: Outliers were defined as values below Q1 − 1.5×IQR or above Q3 + 1.5×IQR.

Abbreviations: IQR, interquartile range; BMI, body mass index; TG, triglycerides; HDL-C, high-density lipoprotein cholesterol; FBG, fasting blood glucose; SBP, systolic blood pressure; DBP, diastolic blood pressure; SCr, serum creatinine; BUN, blood urea nitrogen; eGFR, estimated glomerular filtration rate.

Table A2: Distribution of outliers by metabolic syndrome status.

**MetS**	**No(n/%)**	**Yes(n/%)**
25(OH)D (ng/mL)	419（1.81）	77（1.28）
BMI (kg/m²)	113（0.49）	346（5.76）
Waist (cm)	16（0.07）	57（0.95）
TG (mmol/L)	752（3.24）	906（15.09）
HDL-C (mmol/L)	457（1.97）	27（0.45）
FBG (mmol/L)	892（3.84）	1208（20.13）
SBP (mmHg)	109（0.47）	97（1.62）
DBP (mmHg)	41（0.18）	59（0.98）
SCr_umol(umol)	221（0.95）	134（2.23）
BUN(mmol/l)	443（1.91）	185（3.08）
eGFR (mL/min/1.73 m²)	116（0.50）	80（1.33）
ALT(U/L)	1111（4.79）	1011（16.84）
AST(U/L)	912（3.93）	769（12.81）
Total	23212	6002

Note: Values are presented as number (percentage). Group differences were assessed using the chi-square test.

Abbreviations: MetS, metabolic syndrome; see Table A1 for other abbreviations.

Table A3: E-values for the association between serum 25(OH)D and metabolic syndrome.

Comparison	OR (95% CI)	E-value	E-value (lower 95% CI)
Q2 vs Q1	0.92 (0.83–1.02)	1.04	1.25
Q3 vs Q1	0.79 (0.71–0.88)	1.13	1.51
Q4 vs Q1	0.64 (0.57–0.71)	1.25	1.82

Note: E-values quantify the minimum strength of association that an unmeasured confounder would need to have with both serum 25(OH)D and metabolic syndrome to fully explain away the observed association, conditional on the measured covariates.

Abbreviations: OR, odds ratio; CI, confidence interval.
